# Kinetics of *Mycobacterium tuberculosis*-specific IFN-γ responses and sputum bacillary clearance in HIV-infected adults during treatment of pulmonary tuberculosis

**DOI:** 10.1016/j.tube.2015.05.009

**Published:** 2015-07

**Authors:** David T. Mzinza, Derek J. Sloan, Kondwani C. Jambo, Doris Shani, Mercy Kamdolozi, Katalin A. Wilkinson, Robert J. Wilkinson, Geraint R. Davies, Robert S. Heyderman, Henry C. Mwandumba

**Affiliations:** a Malawi-Liverpool-Wellcome Trust Clinical Research Programme, University of Malawi College of Medicine, P.O. Box 30096, Chichiri, Blantyre 3, Malawi; b Department of Medicine, College of Medicine, Private Bag 360, Chichiri, Blantyre 3, Malawi; c Institute of Infection and Global Health, University of Liverpool, Liverpool L6 1LY, UK; d Department of Clinical Sciences, Liverpool School of Tropical Medicine, Liverpool L3 5QA, UK; e Clinical Infectious Diseases Research Initiative, Institute of Infectious Diseases and Molecular Medicine, University of Cape Town, Rondebosch 7701, Cape Town, South Africa; f MRC National Institute for Medical Research, London NW7 1AA, UK; g Department of Medicine, Imperial College, London SW7 2AZ, UK

**Keywords:** *Mycobacterium tuberculosis*, Antigen-specific immunity, IFN-γ, Pulmonary tuberculosis, HIV infection, Anti-tuberculosis treatment

## Abstract

In HIV-uninfected adults with pulmonary tuberculosis (TB), anti-TB treatment is associated with changes in *Mycobacterium tuberculosis* (Mtb)-specific immune responses, which correlate with sputum bacillary load. It is unclear if this occurs in HIV-infected TB patients. We investigated changes in Mtb-specific immune responses and sputum bacillary clearance during anti-TB treatment in HIV-infected and HIV-uninfected adults with pulmonary TB. Sputum bacillary load was assessed by smear microscopy and culture. Mtb-specific IFN-γ secreting peripheral blood mononuclear cells were enumerated using an ELISPOT assay following stimulation with PPD, ESAT-6 and CFP-10. The baseline frequency of Mtb-specific IFN-γ secreting cells was lower in HIV-infected than HIV-uninfected patients (median PPD 32 vs. 104 Spot Forming Units (SFU), p = 0.05; CFP-10 19 vs. 74 SFU, p = 0.01). ESAT-6-specific IFN-γ secreting cells and sputum bacillary load declined progressively during treatment in both HIV-infected and HIV-uninfected patients. HIV infection did not influence the 2-month sputum culture conversion rate (Odds Ratio 0.89, p = 0.95). These findings suggest that changes in ESAT-6-specific immune responses during anti-TB treatment correspond with changes in sputum bacillary load irrespective of host HIV infection status. The utility of Mtb-specific IFN-γ responses as a proxy measure of treatment response in HIV-infected TB patients warrants further evaluation in other settings.

## 1 Introduction

Tuberculosis (TB) is a leading cause of morbidity and mortality worldwide. Cell-mediated immune responses are pivotal in the host's response to *Mycobacterium tuberculosis* (Mtb) infection and IFN-γ produced predominantly by CD4^+^ T lymphocytes is a crucial component of this response [1–4]. Production of IFN-γ in response to Mtb-specific antigens is commonly used as a marker of potentially protective immunity against Mtb [5–7]. The 20- to 40-fold increased risk of developing active TB among HIV-infected individuals, particularly those with low blood CD4^+^ T lymphocyte counts [8] underscores the importance of T cell-mediated adaptive immunity in protection against disease caused by Mtb.

Successful treatment of pulmonary TB is associated with reduction in sputum bacillary load and assessment of sputum by smear microscopy and culture after two months of anti-TB treatment to monitor early microbiological response is recommended by the International Union Against Tuberculosis and Lung Disease (IUATLD) [9]. Both microbiological assessments, however, have limitations. First, the sensitivity of sputum smear microscopy for acid-fast bacilli (AFB) is low, particularly when the bacillary load is reduced by anti-TB treatment [10]. Second, Mtb grows slowly in culture and may take up to 8 weeks or longer before a positive culture becomes detectable [10]. Other laboratory markers such as C-reactive protein (CRP) and erythrocyte sedimentation rate (ESR) have been used to monitor response to anti-TB treatment [11,12], but they are not specific to TB. Therefore, there is need to identify other laboratory parameters specific to TB which could be useful rapid surrogate markers of response during treatment of pulmonary TB irrespective of the patient's HIV infection status.

In HIV-uninfected adults with pulmonary TB, a good response to anti-TB treatment is associated with changes in peripheral blood immune parameters, including T-cell subpopulations, cellular proliferative and cytokine responses [13–16]. Data from previous studies suggest a correlation between disease activity, bacterial load and IFN-γ production by sensitized lymphocytes in response to Mtb-specific antigens Early-Secreted Antigenic Target 6 (ESAT-6) and Culture Filtrate Protein 10 (CFP-10) during treatment of pulmonary TB [17–22]. How Mtb-specific immune responses relate to microbiological responses during treatment of pulmonary TB in HIV-infected adults is, however, incompletely understood.

We addressed this knowledge gap by conducting a prospective observational cohort study of adults receiving treatment for confirmed pulmonary TB in Malawi where over 50% of adults with sputum smear-positive pulmonary TB are HIV-infected [23]. The aim of the study was to assess the relationship between host Mtb-specific immune responses and sputum bacillary load during the first 2 months of anti-TB treatment in HIV-infected pulmonary TB patients.

## 2 Materials and methods

### 2.1 Study population

HIV-infected and HIV-uninfected adults aged ≥17 years were recruited at the Queen Elizabeth Central Hospital (QECH) in Blantyre, Malawi. Participants were patients with microbiologically confirmed pulmonary TB whose Ziehl–Neelsen (ZN)-stained sputum smears were graded ≥1+ positive for AFB at direct microscopy [10]. Asymptomatic volunteers with no clinical evidence of active disease or previous history of TB treatment were also recruited from communities surrounding QECH as controls. All participants were BCG-vaccinated at birth. Patients were recruited before commencing anti-TB treatment and were followed up to 56 days of treatment, while controls were seen once at recruitment. Anti-TB treatment was given as short course chemotherapy consisting of rifampicin, isoniazid, pyrazinamide and ethambutol for 2 months (intensive phase), followed by rifampicin and isoniazid for 4 months (continuation phase) according to national guidelines. Written informed consent was obtained from all study participants and the research ethics committees of the Malawi College of Medicine (COMREC) and the Liverpool School of Tropical Medicine approved the study.

### 2.2 Sample collection

Peripheral blood and sputum samples were collected from patients before and after 14, 28 and 56 days of anti-TB treatment. Peripheral blood was collected from controls at recruitment only.

### 2.3 Processing of sputum for mycobacterial culture and detection of Mtb

Sputum samples digested and decontaminated by the N-acetyl *l*-cysteine (NALC) (Sigma–Aldrich, Germany) and 3% sodium hydroxide (NaOH) (VWR, Belgium) method were processed for mycobacterial culture using the Bactec™ MGIT™ 960 system (Becton Dickinson, USA) as previously described [24]. Days to positivity (DTP), defined as the time it took for MGIT cultures to become positive were used as an inverse measure of the bacillary load in the sputum sample. Smears were prepared from positive MGIT cultures, stained with ZN stain and examined for AFB by light microscopy. The presence of Mtb in all cultures that were positive for AFBs was confirmed using the MPT64 antigen test (Becton Dickinson, USA) according to the manufacturer's instructions. MGIT cultures were positive for Mtb if both smear microscopy for AFB and MPT64 antigen test results were positive. They were reported negative if there was no growth after 42 days incubation.

### 2.4 Antigens and enumeration of Mtb-specific IFN-γ secreting cells

Peripheral blood mononuclear cells (PBMCs) were isolated from heparinised blood using the gradient centrifugation technique as previously described [25]. Freshly isolated PBMCs were analysed for IFN-γ production using an 18-hour enzyme-linked immunospot (ELISPOT) assay as described elsewhere [26]. Antigens/peptides were added individually to duplicate wells containing 0.25 × 10^6^ PBMCs at 10 μg/ml purified protein derivative (PPD) (Statens Serum Institute, Copenhagen, Denmark), 5 μg/ml CFP-10, 5 μg/ml ESAT-6 (Peptide and Protein Research, UK) and 5 μg/ml phytohaemagglutinin (PHA) (Sigma–Aldrich, Germany) as the positive control. Additional duplicate wells left unstimulated were the negative control. Spot Forming Units (SFU) were quantified using an automated ELISPOT reader (AID Autoimmune Diagnostic GmbH, Strassberg, Germany), and data were expressed as SFU per million PBMCs. Control values (from unstimulated wells) were subtracted from antigen–stimulated conditions, and responses were scored as positive if antigen-stimulated wells contained ≥ 5 SFU more than unstimulated wells as described previously [27].

### 2.5 Statistical analysis

Data analysis and graphical presentations were performed using GraphPad Prism 5 (GraphPad Software, USA). Non-paired comparisons were done either by Mann–Whitney U or Kruskall Wallis and Dunn's multiple comparison tests. Logistic regression was used to determine the kinetics of bacillary clearance, while One-way ANOVA was used to determine the kinetics of Mtb-specific immune responses over time. Linear regression was used to determine the association between CD4 count and Mtb-specific immune responses. Results are given as medians with inter-quartile ranges (IQR). Differences were considered statistically significant when p < 0.05.

## 3 Results

### 3.1 Participant characteristics

We recruited 63 sputum smear- and culture-positive pulmonary TB patients and 27 asymptomatic controls (Table 1). Among HIV-infected patients 44% (12/27) had a CD4^+^ T-cell count of ≤200 cells/μl at recruitment. All participants on antiretroviral therapy (ART) received the first-line regimen for Malawi at the time of the study consisting of stavudine, lamivudine and nevirapine. Nine ART-naïve patients commenced ART within 2 weeks of starting anti-TB treatment according to national guidelines. All patients had good clinical response to anti-TB treatment during the study period. Chest radiographs were not repeated to monitor response to treatment during follow-up unless clinically indicated by worsening respiratory symptoms and signs. ESR, CRP and plasma HIV viral load were not monitored during the study.

### 3.2 HIV infection is not associated with failure to clear Mtb from sputum during the intensive phase of anti-TB treatment

**Figure 1:**
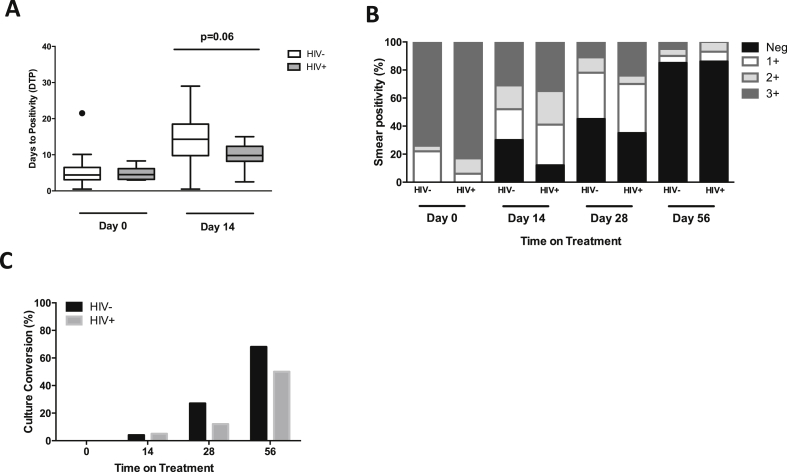
Sputum conversion in HIV-infected and HIV-uninfected pulmonary TB patients on anti-TB treatment. Sputum samples were collected from TB patients before (baseline), and at days 14, 28 and 56 of anti-TB treatment. The grade of smear positivity at direct microscopy and days to positivity (DTP) for MGIT liquid cultures was used as a surrogate measure of bacterial load in sputum samples. DTP inversely correlates with the bacillary load. Culture conversion was calculated by determining the proportion of patients whose sputum samples did not grow bacilli in culture or whose sputum smear microscopy was negative for acid-fast bacilli. A) Comparison of bacillary load in sputum samples from HIV-infected and HIV-uninfected TB patients before and after 14 days of anti-TB treatment (Day 0 HIV− n = 23, HIV+ n = 19; Day 14 HIV− n = 22 HIV+ n = 18). Data were analysed using the Mann–Whitney U test; black horizontal bars represent medians and IQRs. B and C) Sputum smear and culture conversion in HIV-infected and HIV-uninfected patients during the intensive phase of anti-TB treatment (Day 0 HIV− n = 23, HIV+ n = 19; Day 14 HIV− n = 23 HIV+ n = 19; Day 28 HIV− n = 19, HIV+ n = 18; Day 56 HIV− n = 19, HIV+ n = 16). The bars represent medians.

To assess the effect of HIV on microbiological responses, we determined the sputum bacillary load before, and at days 14, 28 and 56 of anti-TB treatment. The grade of smear positivity and the DTP of MGIT cultures were used as surrogate measures of sputum bacillary load. Before commencing anti-TB treatment, HIV-infected and HIV-uninfected patients had similar sputum bacillary loads (grade 3+ smear positivity 83% vs. 74%; p = 0.339, median DTP 4.4 [IQR 3.1–6.5] days vs. 4.5 [IQR 3.2–6.2] days; p = 0.98) (Figure 1A and 1B). After 14 days of treatment the magnitude of bacillary load reduction was greater in HIV-uninfected than HIV-infected patients, although this did not reach statistical significance for culture (median DTP 14.3 [IQR 9.8–18.5] days vs. 9.8 [IQR 8.2–12.4] days, p = 0.06, Figure 1A) but was significant for smear conversion (30% vs. 12%, p = 0.0029, Figure 1B). However, the proportion of patients who successfully cleared Mtb after 2 months of treatment was similar between the two groups (Day 56 smear conversion, HIV− 85% vs. HIV+ 86%, Figure 1B; culture conversion, HIV− 68% vs. HIV+ 50%, Figure 1C; OR 0.89 [IQR 0.02–32.1], p = 0.95). While these findings suggest that clearance of Mtb from sputum very early during anti-TB treatment may be slower in HIV-infected than HIV-uninfected individuals, HIV infection is not associated with failure to clear bacilli as duration of anti-TB treatment increases.

### 3.3 HIV-infected TB patients have low frequency of PPD- and CFP-10 specific IFN-γ secreting cells before commencing anti-TB treatment

**Figure 2:**
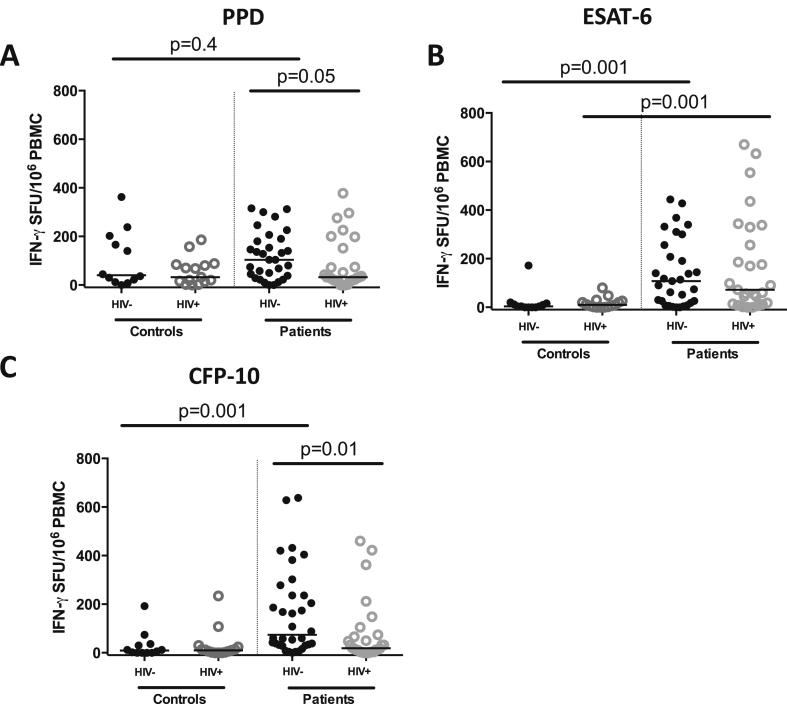
IFN-γ responses to Mtb-specific antigens in HIV-infected and HIV-uninfected asymptomatic controls and TB patients before commencing anti-TB treatment. Peripheral blood mononuclear cells (PBMCs) were stimulated with PPD, ESAT-6, CFP-10 or PHA in an 18 h ELISPOT assay. PHA stimulation was used as the positive control while unstimulated cells were the negative control. The frequencies of A) PPD-specific, B) ESAT-6-specific and C) CFP-10-specific IFN-γ secreting cells were compared between HIV-infected and HIV-uninfected TB patients and asymptomatic controls. Data were analysed using Kruskal–Wallis and Dunn's multiple comparisons tests; black horizontal bars represent medians after background (unstimulated) responses were subtracted from all the antigen-specific responses (Controls, HIV− n = 12, HIV+ n = 15; TB Patients, HIV− n = 31, HIV+ n = 27).

Next, we compared the Mtb antigen-specific IFN-γ responses between HIV-infected and HIV-uninfected TB patients before the start of anti-TB treatment. The frequency of IFN-γ secreting cells following *ex vivo* stimulation of PBMCs with PPD, ESAT-6 and CFP-10 was measured using an ELISPOT assay. We found that the frequency of PPD- and CFP-10-specific IFN-γ secreting cells was lower in HIV-infected than HIV-uninfected patients (median PPD 32 [IQR 14–84] vs. 104 [IQR 38–190] SFU, p = 0.05; median CFP-10 19 [IQR 4–72] vs. 74 [IQR 32–247] SFU, p = 0.01; Figure 2A and 2B). There was no significant difference in the frequency of ESAT-6-specific IFN-γ secreting cells between the two groups (median ESAT-6 72 [IQR 14–330] vs. 108 [IQR 18–256], p = 0.8923). The number of antigen-specific IFN-γ secreting cells did not correlate with the blood CD4^+^ T-cell count at recruitment (PPD, r^2^ = 0.004, p = 0.77; CFP-10, r^2^ = 0.04, p = 0.32) suggesting that the blunted IFN-γ responses may reflect a preferential loss or dysfunction of PPD- and CFP-10-specific CD4^+^ T cells in HIV-infected individuals as reported previously [28,29].

To determine if the antigen-specific responses observed in TB patients were due to active TB, we compared ELISPOT responses between TB patients and asymptomatic controls. The frequency of ESAT-6- and CFP-10-specific IFN-γ secreting cells was higher in HIV-uninfected TB patients than HIV-uninfected controls (median ESAT-6108 [IQR 18–256] vs. 4 [IQR 0–15] SFU, p = 0.01; median CFP-10 74 [IQR 32–247] vs. 9 [IQR 0–35] SFU, p = 0.001; Figure 2B and 2C). The frequency of PPD-specific IFN-γ secreting cells was not significantly different between HIV-uninfected patients and controls (median PPD 104 [IQR 38–190] vs. 40 [IQR 15–193], p = 0.4255). ESAT-6-specific responses were also higher in HIV-infected TB patients compared to HIV-infected controls (median ESAT-6 72 [IQR 14–330] vs. 10 [IQR 2–26] SFU, p = 0.001; Figure 2C). The frequencies of PPD- and CFP-10-specific responses were not significantly different between HIV-infected patients and controls (median PPD 32 [IQR 18–152] vs. 32 [IQR 14–84], p = 0.7015; median CFP-10 19 [IQR 4–72] vs. 10 [IQR 2–24], p = 0.1155). These findings suggest that high ELISPOT responses to Mtb-specific antigens ESAT-6 and CFP-10 in TB patients reflect high bacillary loads during active disease. They also suggest that HIV infection may differentially impact host Mtb-specific responses, with CFP-10-specific responses affected more than ESAT-6-specific responses. The absence of a significant difference in PPD-specific responses between patients and controls may be due to previous BCG vaccination and comparable exposure to environmental mycobacteria.

### 3.4 Kinetics of PPD-, ESAT-6- and CFP-10-specific IFN-γ secreting cells during the intensive phase of anti-TB treatment

**Figure 3:**
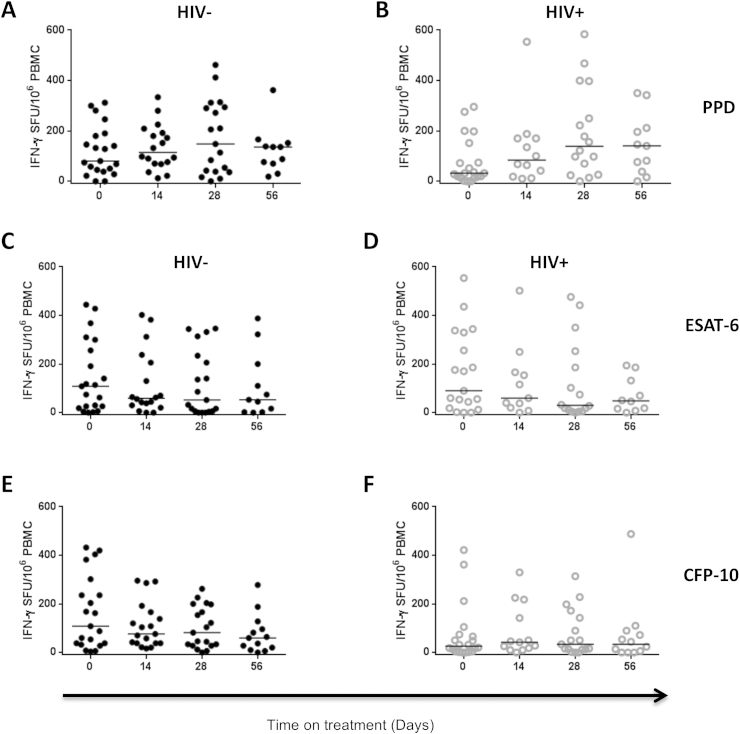
Kinetics of PPD-, ESAT-6- and CFP-10-specific IFN-γ responses during the intensive phase of anti-TB treatment. Peripheral blood was collected from TB patients before (day 0) and at days 14, 28 and 56 of anti-TB treatment. Peripheral blood mononuclear cells (PBMCs) were stimulated with PPD, ESAT-6, CFP-10 or PHA in an 18 h ELISPOT assay. PHA stimulation was used as the positive control and unstimulated cells were used as the negative control. The frequency of PPD-, ESAT-6- and CFP-10-specific IFN-γ secreting cells at different time-points during the intensive phase of anti-TB treatment are shown for both HIV-uninfected and HIV-infected TB patients. The frequency of PPD-specific IFN-γ secreting cells in (A) HIV-uninfected and (B) HIV-infected TB patients (HIV− Day 0 n = 21, Day 14 n = 18, Day 28 n = 19, Day 56 n = 11; HIV+ Day 0 n = 20, Day 14 n = 12, Day 28 n = 16, Day 56 n = 11). The frequency of ESAT-6-specific IFN-γ secreting cells in (C) HIV-uninfected and (D) HIV-infected TB patients (HIV− Day 0 n = 21, Day 14 n = 18, Day 28 n = 19, Day 56 n = 11; HIV+ Day 0 n = 19, Day 14 n = 11, Day 28 n = 16, Day 56 n = 10). The frequency of CFP-10-specific IFN-γ secreting cells in (E) HIV-uninfected and (F) HIV-infected TB patients (HIV− Day 0 n = 20, Day 14 n = 19, Day 28 n = 19, Day 56 n = 13; HIV+ Day 0 n = 20, Day 14 n = 13, Day 28 n = 17, Day 56 n = 12). Data were analysed using One-way ANOVA; black horizontal bars represent medians after background (unstimulated) responses were subtracted from all the antigen-specific responses.

We examined changes in Mtb antigen-specific IFN-γ responses during the first 2 months of anti-TB treatment by comparing the frequencies of IFN-γ secreting cells before (day 0) and after 14, 28 and 56 days of treatment in HIV-infected and HIV-uninfected TB patients. Although there were no statistically significant differences between day 0 and day 56 in responses to PPD (HIV−, p = 0.6888; HIV+, p = 0.0846), ESAT-6 (HIV−, p = 0.1220; HIV+, p = 0.9923) or CFP-10 (HIV−, p = 0.4508; HIV+, p = 0.2417), the frequency of PPD-specific IFN-γ secreting cells showed an increasing trend from day 0 to day 56 in both HIV-infected and HIV-uninfected patients (Table 2, Figure 3A and 3B). In contrast, the frequency of ESAT-6-specific IFN-γ secreting cells showed a decreasing trend from day 0 to day 56 in both HIV-infected and HIV-uninfected patients (Table 2, Figure 3C and 3D). There was no clear trend in the frequency of CFP-10-specific IFN-γ secreting cells in both HIV-infected and HIV-uninfected patients (Table 2, Figure 3E and 3F). These findings suggest similar kinetics of PPD-, ESAT-6- and CFP-10-specific IFN-γ secreting cells during anti-TB treatment between HIV-infected and HIV-uninfected TB patients although the trends in responses differ between antigens. Differences in antigen loads during treatment may underlie the different trends in antigen-specific responses.

## 4 Discussion

The present study investigated the kinetics of Mtb-specific immunity and sputum microbiological responses during the intensive phase of anti-TB treatment in adults with pulmonary TB. We have shown that while HIV infection may be associated with slow sputum bacillary clearance very early in the course of anti-TB treatment, it did not impact the sputum conversion rate after 2 months of treatment. These findings are consistent with, and advance what was reported previously that HIV infection does not influence the time to sputum conversion in sputum smear-positive pulmonary TB patients [30]. Furthermore, HIV-infected patients had lower frequencies of PPD- and CFP-10-specific IFN-γ secreting cells before the start of anti-TB treatment than HIV-uninfected patients, perhaps due to loss or dysfunction of Mtb-specific CD4^+^ T-cells [28,29]. Together, the findings imply that HIV-associated impaired host Mtb-specific immune responses before commencing anti-TB treatment do not predict subsequent microbiological failure.

The RD1-coded antigens ESAT-6 and CFP-10 are specific to Mtb and were reported to be more specific than PPD for detecting Mtb in HIV-uninfected patients with active TB [31]. Consistent with this report, we found a higher frequency of ESAT-6- and CFP-10-specific IFN-γ secreting cells in HIV-uninfected TB patients than HIV-uninfected controls. In contrast, only ESAT-6-specific IFN-γ secreting cell responses were significantly different between HIV-infected TB patients and HIV-infected controls, suggesting a differential impact of HIV infection on Mtb antigen-specific immune responses. CFP-10-specific responses appear to be influenced more by HIV infection than ESAT-6-specific responses. This observation underscores the previously reported limited accuracy of RD1-coded Mtb antigen-specific immune responses for diagnosing active TB in HIV-infected individuals [32].

During the first 2 months of anti-TB treatment the frequency of ESAT-6-specific IFN-γ secreting cells showed a decreasing trend with increasing duration of treatment in both HIV-infected and HIV-uninfected patients while the opposite was true for PPD-specific responses. Other studies have reported similar trends in the kinetics of cytokine responses to PPD, ESAT-6 and CFP-10 in HIV-infected and HIV-uninfected children [33,34] and HIV-uninfected adults during treatment of pulmonary TB [16]. While the reasons for this dichotomy are not clear, we speculate that the decline in Mtb antigen-specific immune responses may be explained by a decline in antigen load as a consequence of successful anti-TB treatment since these proteins are secreted by live and actively metabolizing bacilli [35]. The differences may also reflect inadequate expansion of a small reservoir of ESAT-6- and CFP-10-specific CD4^+^ T cells during anti-TB treatment [36,37]. In contrast, the increase in the frequency of PPD-specific immune responses with increasing duration of anti-TB treatment may reflect an appropriate response to an increasing load of mycobacterial proteins released by dying bacilli early during the intensive phase of treatment. The increase in PPD-specific responses may also be due to rapid expansion of a large reservoir of PPD-specific CD4^+^ T cells established following previous BCG vaccination and exposure to environmental mycobacteria [36,37]. Although our study did not look beyond the first two months of treatment, others have shown that the PPD-specific IFN-γ response eventually wanes after three to six months of anti-TB treatment [16,34].

This study had limitations. First, the number of patients recruited was small, raising the possibility that small differences in immune responses between HIV-infected and HIV-uninfected patients may have been missed. Second, patients were followed up for the first 2 months of anti-TB treatment only, so it was not possible to link changes in immune and microbiological parameters during the follow up period with outcomes at the end of 6 months of treatment.

## 5 Conclusion

This study has shown that HIV infection does not influence the 2-month sputum smear and culture conversion rate in HIV-infected adults receiving first-line treatment for pulmonary TB. The frequency of ESAT-6-specific IFN-γ secreting cells declines progressively during anti-TB treatment and corresponds with declining sputum bacillary load in both HIV-infected and HIV-uninfected patients. Serial measurements of ESAT-6-specific IFN-γ secreting cells may be useful for monitoring response to anti-TB treatment irrespective of host HIV infection status. Large studies in different populations are required to determine further the potential utility of Mtb antigen-specific immune responses as a proxy measure of anti-TB treatment response and outcome in HIV-Infected and HIV-uninfected adult pulmonary TB patients.

## Author's contributions

Study conception and design: DTM, HCM, DJS, RSH, GRD, RJW, KAW; Data acquisition, analysis and interpretation: DTM, KCJ, HCM, DJS, GRD, DS, MK; Drafting and revising the manuscript for important intellectual content: HCM, DTM, KCJ, DJS, RSH, GRD, RJW, KAW; Final approval: DTM, DJS, KCJ, DS, MK, KAW, RJW, GRD, RSH, HCM.

## Figures and Tables

**Table 1:** Characteristics of study participants.

	Pulmonary TB patients	Asymptomatic controls
Total	63	27
Age, median (IQR)	30 (25–36)	33 (29–39)
Sex		
Male (%)	45 (71)	12 (44)
HIV Status		
HIV+ (%)	27 (43)	15 (56)
HIV+ on ART (%)	18 (67)	10 (67)
Baseline CD4, median (IQR)∗	17 (68–287)	452 (237–610)

∗ CD4^+^ T-cell counts were done in HIV-infected individuals only; ART = antiretroviral therapy; IQR = interquartile range.

**Table 2:** Mtb antigen-specific IFN-γ responses in pulmonary TB patients during the first 2 months of anti-TB treatment.

Days on treatment	Median PPD-specific SFU [IQR]		Median ESAT-6-specific SFU [IQR]		Median CFP-10-specific SFU [IQR]	
	HIV−	HIV+	HIV−	HIV+	HIV−	HIV+
0	80 [42–185]	32 [15–133]	108 [22–278]	90 [18–330]	108 [35–269]	26 [4–72]
14	115 [70–196]	84 [24–170]	59 [28–214]	60 [20–166]	76 [38–166]	42 [14–180]
28	138 [38–294]	139 [37–361]	52 [6–214]	30 [8–236]	82 [28–198]	34 [13–158]
56	136 [70–152]	140 [38–212]	53 [2–200]	48 [14–146]	60 [15–112]	34 [15–86]

SFU = spot forming units; IQR = interquartile range.
